# Challenges encountered in providing integrated HIV, antenatal and postnatal care services: a case study of Katakwi and Mubende districts in Uganda

**DOI:** 10.1186/s12978-016-0162-8

**Published:** 2016-04-18

**Authors:** Sharon Eva Ahumuza, Joseph Rujumba, Abdallah Nkoyooyo, Raymond Byaruhanga, Rhoda K. Wanyenze

**Affiliations:** MakSPH-CDC Fellowship Program, Makerere University College of Health Sciences, School of Public Health, P.O. Box 7072, Kampala, Uganda; Department of Peadiatrics and Child Health, College of Health Sciences, School of Medicine, Makerere University, P.O. Box 7072, Kampala, Uganda; AIDS Information Center, Plot 1321 Musaja Alumbwa, Mengo-Kisenyi, Kampala, Uganda; Department of Disease Control and Environmental Health, Makerere University College of Health Sciences, School of Public Health, P.O. Box 7072, Kampala, Uganda

**Keywords:** Integrated HIV, Antenatal services, Postnatal service and challenges in providing integrated services

## Abstract

**Background:**

Integration of sexual and reproductive health (SRH), HIV/AIDS and maternal health (MH) services is a critical strategy to confront the HIV/AIDS epidemic, high maternal mortality and the unmet need for contraception. In 2011 the AIDS Information Centre (AIC) in partnership with the Ministry of Health implemented SRH, HIV/AIDS and MH integration services in the districts of Katakwi and Mubende in Uganda. This paper documents challenges encountered in providing these integrated services in the two districts.

**Methods:**

This was a cross-sectional qualitative study conducted in Mubende and Katakwi districts in Uganda. Data were collected using 10 focus group discussions with 89 women attending ANC and postnatal care and 21 key informant interviews with district managers and health workers who were involved in the integrated service delivery. Content thematic approach was used for data analysis.

**Results:**

The study findings indicate that various challenges were encountered in integrating HIV, ANC and PNC services. Major challenges included inadequate staff, gaps in knowledge of service providers especially with regard to provision of long-term family planning, limited space, shortage of critical supplies such as HIV test kits, drugs and gloves.

**Conclusion:**

These findings indicate that the delivery of integrated HIV, SRH and MH services is hampered greatly by health system challenges and depict the need for additional staffing in health facilities, capacity building of health workers and health managers as well as ensuring sufficient supplies to health facilities for smooth implementation of integrated SRH, HIV and MH services.

## Background

Integration of Sexual and reproductive health (SRH), HIV/AIDS and maternal health (MH) services, is a critical strategy to confront the inter-connected public health challenges of HIV/AIDS, high maternal mortality and the unmet need for contraception [1, 2], especially in high HIV prevalence settings. Integrated services enhance uptake of both SRH and HIV services [3–5] and are more convenient as they minimize referrals and save time for the patients. Integration also helps in preventing unwanted pregnancies and prenatal transmission of HIV by extending contraceptives and other family planning (FP) services to women of reproductive age [6, 7]. Further, integration reduces HIV related stigma and discrimination and gender based violence thus increasing uptake of HIV/AIDS and SRH services [8]. Some of the challenges to integration of services include; infrastructure such as multiple and ill equipped consultation rooms, negative attitude of health workers (HW), poor communication, high patient load, and limited skills of HWs [5].

The Uganda national strategy on SRH, HIV/AIDS and MH service integration provides for horizontal integration of HIV/AIDS and SRH services by ensuring that persons living with HIV (PLHIV) receive HIV prevention, care and treatment and FP services [9]. In 2010, the AIDS Information Centre (AIC) in collaboration with MoH piloted an SRH, HIV/AIDS and MH integration model to promote provider-initiated service provision, where the health facility avails information on services and the clients either accept services or opt out. The model emphasized provision of SRH, HIV/AIDS and MH services at all points in health facilities from health center III to general hospitals. Due to resource constraints, this assessment focused on the segment integrating HIV services into ANC and PNC services at health center IV and district hospitals. In addition, intrapartum care was not assessed as it was not part of the model.

**Fig. 1:**
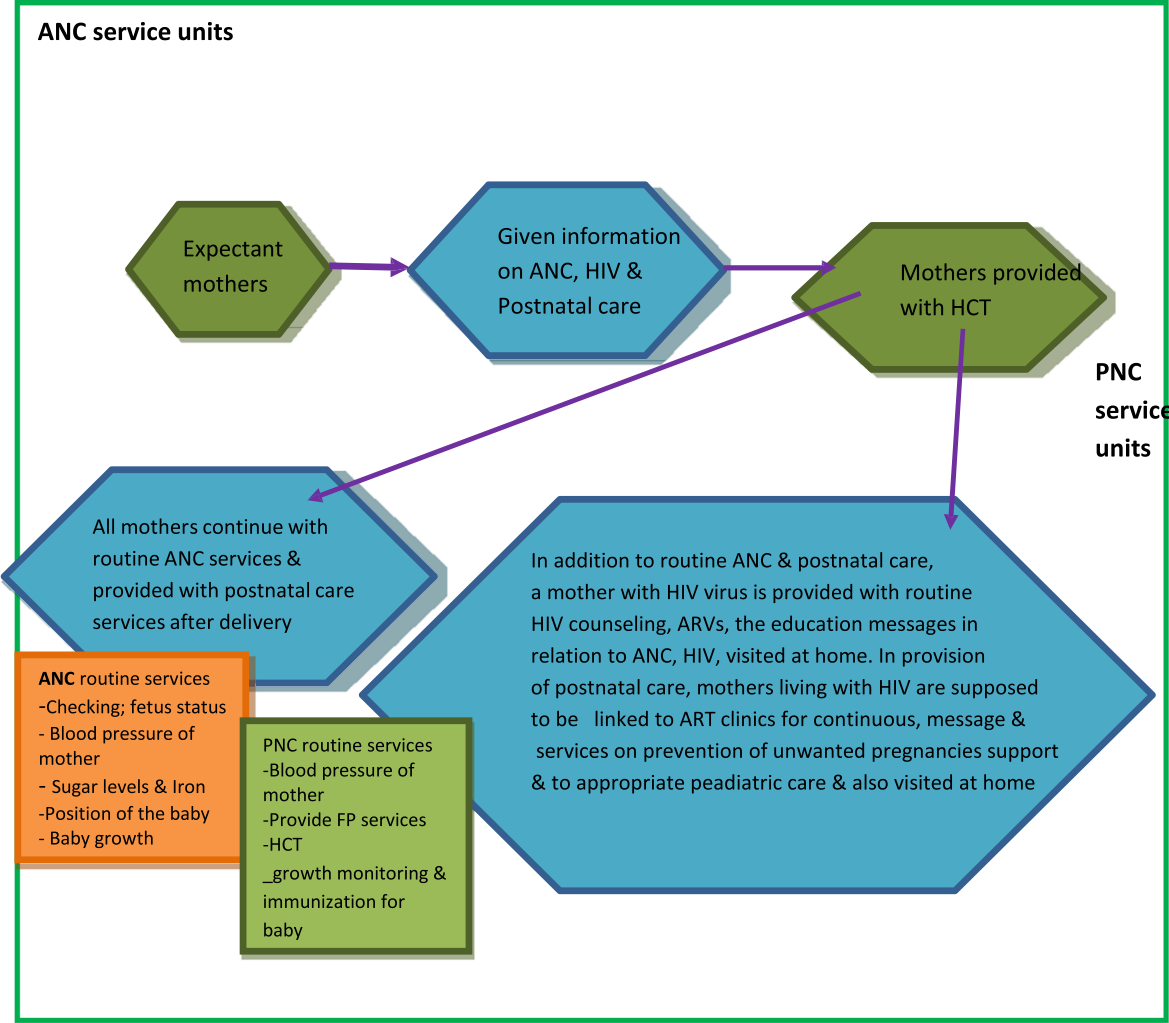
A simple flow chart to illustrate what was expected to be provided to expectant mothers in integrating HIV into ANC and postnatal care services at district hospitals and health center IVs

As shown in Fig. 1, all expectant women who visited ANC clinics, should be given information on ANC, PNC, and HIV prevention including prevention of mother to child transmission (PMTCT). Similarly all mothers should be offered HIV counseling and testing. HIV positive women should be provided with ongoing HIV counseling, ARVs, education messages in relation to ANC, PNC and HIV, visited at home, provided with information and services on prevention of unwanted pregnancies, linked to ART clinics for continuous support and to appropriate peadiatric care for the infants [10]. However, the implementation of this model has not been assessed. The aim of the study was to explore the challenges encountered in providing integrated HIV, antenatal and postnatal care services in Katakwi and Mubende districts. Integration in this paper denotes the provision of comprehensive HIV, ANC and PNC services at the same health facility and through referrals from one service provider to another [11]. In this regard, integration implies that clients receive general health information and HIV, ANC and PNC services based on need across different levels of the health system.

## Methods

### Study design

This was a descriptive cross-sectional qualitative study. A qualitative design was chosen to facilitate an in-depth exploration of the challenges from the perspectives of service users, providers and district managers [12, 13]. Data collection took place from May to June, 2014.

### Research site and setting

The study was carried out in Katakwi and Mubende districts, the first two districts to pilot the AIC integrated model. General hospitals and level IV health centers were the focus of the study as they were expected to provide integrated SRH, HIV and maternal health services for in and out patients. Due to resource constraints the study could not be extended to level III health centers. Katakwi district, located in Eastern Uganda, has one health center at level IV and one general hospital. Mubende, located in Central Uganda, has two health centers level IV and one government general hospital [14]. All the level IV and general hospitals (five health facilities) in the two districts were included in the study.

### Study participants and selection procedure

The study participants included the district health team (DHT) members, health care providers (KIs) and service users (FGD participants) at 2 district hospitals (1 from each of the study districts) and 3 health center level IV (1 in Katakwi and 2 in Mubende district). Overall, 21 key informant interviews were conducted with; 2 District Health Officers (DHOs), 2 district SRH officers and 2 district HIV focal persons, 2 Hospital Directors, 1 Obstetrician and 3 midwives/nursing officers in ANC and PNC units. At health center IVs, the KIs included 3 health center IV in charges and 6 nursing officers in ANC and PNC. Participants were purposively selected based on their involvement in the integrated service delivery. Women attending ANC and PNC participated in Focus group discussions. A total of 10 focus group discussions (FGDs) with 89 women, each comprising of 6–10 members were conducted. The FGD participants were 18 years and above and had attended at the same health facility more than once to enable them have an understanding of the services provided and challenges encountered.

### Data collection

FGDs were conducted with service users to enlist their experiences with service integration and the challenges they encountered. The FGDs were composed of the mothers receiving ANC and PNC services. Overall, 4 FGDs (2 for ANC and 2 for PNC) were conducted at district hospitals and 6 FGDs (3 for ANC and 3 for PNC) at HC level IV. An FGD guide was used to conduct the interviews. The FGDs explored; type of services provided at the health facilities, the challenges encountered in utilization of integrated HIV, ANC and PNC services and suggestions for improvement. FGDs were conducted in Ateso and Luganda, the dominant languages in Katakwi and Mubende Districts respectively. Two researchers (a facilitator and a note taker) conducted the FGDs which were audio recorded and transcribed.

For the interviews with district health managers and health care providers, a key informant interview guide was used to explore the challenges encountered during integrated service provision. Interviews also sought for suggestions to improve the integrated service delivery. The interviews were conducted in English by a facilitator and a note taker. Interviews were audio recorded and transcribed. Interview guides were pre-tested to ensure that they generated the required information. Results from this phase were excluded from final analysis.

### Data management and analysis

The researcher trained data collectors on the objectives of the study and the process of data collection and management. Recordings of interviews were simultaneously translated and transcribed using Microsoft word. Two people transcribed and translated the interviews and compared notes to check for consistency of translation. Data analysis was done manually. This involved reading scripts several times and generating the major themes and sub-themes which were used to code the data. Direct quotations were identified and used in presentation of study findings. The identities of individual study participants were masked, for instance we used KI or FGD, Katakwi or Mityana district to mean study participants from the two districts. The key themes were challenges encountered by health care providers during the provision of HIV care with ANC and PNC and suggestions for improvement.

### Ethical consideration

Ethical clearance was obtained from Makerere University School of Public Health Higher Degrees Research and Ethics Committee and the National Council for Science and Technology. Clearance was also obtained from the AIDS Information Center, district and facility managers. Written informed consent was obtained from each of the study participants before conducting interviews. To ensure confidentiality, respondents were identified using codes.

## Results

### Socio-demographic characteristics of mothers

Table 1 shows the characteristics of the 89 mothers who participated in FGDs. Majority of the participants (58 %) were between 18 and 24 years old, while only (4 %) were above 35 years. Most participants (90 %) were married and all (100 %) had ever attended school; 58 % primary level and 40 % secondary level. Most participants were involved in farming (87 %) and very few (3 %) were in formal employment. Fifty six percent of the mothers had children below 5 years old (Table 1).

**Table 1:** Socio-demographic characteristics of mothers

	*N* = 89	%
Age		
18–24	51	58
25–30	27	30
31–35	7	8
36+	4	4
Total	89	100
Marital status		
Married	80	90
Single	9	10
Total	89	100
Level of education		
Primary	52	58
Secondary	36	40
Tertiary	1	1
Total	89	100
Type of employment		
Farmer	77	87
Business	7	8
Health worker	2	2
Tailor	1	1
Teacher	1	1
Waitress	1	1
Total	89	100
Mothers with children under 5 years	50	56 %

### Characteristics of the key informants

Out of the 21 KIs, 13 (62 %) were from Mubende District while 8 (38 %) were from Katakwi District. Of the 13 KIs from Mubende District, 5 (38 %) were medical doctors, 1 (8 %) was a medical assistant, 4 (31 %) were midwives and 3 were nurses. In Katakwi District, 3 (37 %) of the KIs were medical doctors, 4 (50 %) were midwives and 1 (13 %) was a clinical officer. Overall, 55 % of the KIs were female.

### Challenges encountered in providing integrated HIV, ANC and PNC services in Katakwi and Mubende districts

The major challenges affecting integrated delivery of HIV, ANC and PNC services were; few staff and staff absenteeism, gaps in knowledge of service providers, inadequate space andshortage of critical supplies. The challenges in each of these themes are further described below.

### Few staff and staff absenteeism

The major challenge affecting integration mentioned by district health managers and service providers was limited staffing compared to the high number of clients in maternal health clinics. The limited staffing constrained the provision of various services for mothers and their babies. This also affected the quality of health services including long waiting time and failure by health workers to provide mothers with critical information needed to promote their health and that of their babies. For example, at some health facilities, vital measurements such as monitoring blood pressure and weighing children were reportedly not done consistently. In the District referral hospitals, there was only one Gyneoncologist and whenever he was not available, final decisions on complex issues could be taken by only midwives.

*“We have only one doctor at the hospital. This makes us (midwives) the final decision makers with no one to consult when he is not available. At times we consult each other and decide on what to do as midwives. When we fail, then we refer the mother to another hospital in a different District, for further care” (Female KIs, Mubende District).*

*“We have few staff expected to provide various services to mothers in ANC & PNC units. We decided to be seeing only70 mothers per day…, but still it is very tiring. In most cases, we fail to provide some of expected services …e.g. weight taking of mothers & babies,” (Female, KI, Katakwi District).*

Staffing challenges were also highlighted in relation to laboratories with most of the health facilities having only one laboratory technician. Health workers noted that whenever a laboratory technician was absent, clients would be attended to without conducting laboratory investigations. Absenteeism of health workers was attributed to various factors but mostly to delay in payment of salaries.

*“We have one laboratory technician who has spent some time without a monthly salary and it is now over 4 months since he stopped working…”(Female KI, ANC Mubende District).*

### Knowledge and skills gaps of health workers

Health workers expressed various knowledge gaps in providing integrated services. The gaps included; administering long-term family planning methods such as intrauterine devices (IUD), and permanent methods and HIV counseling and testing.

“*We have long term methods of FP in our cupboard but none of us (health workers) knows how to administer them. We wait for health workers from Maristopes Uganda who are experienced in administering long term and permanent FP methods” (KI, health facility, Katakwi District).*

*“It is not easy for us to conduct HIV counseling and testing for mothers. It becomes even very difficult for us to give positive results to mothers. Thank God we have the volunteers who are facilitated by Baylor Uganda to provide counseling and testing services, otherwise, we would not have managed…” (KI, Katakwi District).*

The limited knowledge of health workers to administer some of the long term family planning methods was also reported by mothers.

*“The long term family planning methods are available in our health facility but our nurses do not know how to administer them…. So those in need have to wait for health workers from Mari Stopes Uganda come to conduct outreaches” (PNC-FGD participant, Katakwi District).*

### Client-provider relationship

Some health workers mentioned that some of their colleagues force expectant mothers to swallow medicine especially de-worming tablets and were rude to them. Due to fear of non-adherence by mothers with regard to de-worming tablets, health workers instruct them to swallow the tablets at the health facility. To mothers, forcing them to take medicine was very unbecoming. Whereas direct observed therapy is an approach of drug administration being used in many settings in Uganda, inadequate information from the health workers was a source of misunderstanding among mothers.

*“We are forced to swallow de-worming tablets under the watch of health workers. If one refuses, she is not given her passport back (medical records book) and is denied other services….” (FGD, ANC, Katakwi District).*

### Lack of confidentiality

In addition to communication problems, ensuring confidentiality for HIV infected mothers at the health facilities was not adhered to. Ensuring confidentiality was a challenge given the practice of separating the HIV positive mothers from their HIV negative counterparts. The challenges of observing confidentiality was also highlighted by mothers who noted that all ANC attendees were asked to test for HIV after attending general health education talks. Those found to be HIV positive were taken for detailed post-test HIV counseling in a separate room. In almost all the health facilities, the ART and EID rooms were situated next to the ANC and PNC service areas. Health workers call out some mothers’ names and refer them to EID and ART units which make the rest of the mothers suspect or even know their positive sero-status. As a result, HIV infected mothers would feel stigmatized and fear returning to the health facilities.

*“The way nurses separate the positive mothers from us makes the rest to know their HIV status. Nurses just come and read out a name or names of mothers and refer them to ART units for further counseling. Some of the mothers would refuse to go to the ART clinic and would just go home…” (FGD, ANC, Mubende District).*

Indeed, interviews with health care providers confirmed that in some instances actions of health workers could be a basis for some mothers guessing the HIV status of their colleagues. On probing, most health workers mentioned that the practice of separating HIV positive and negative mothers was not meant to reveal their status but to link HIV positive women to appropriate care.

Both providers and users in the two study districts noted that stigmatization of positive mothers discouraged some of the mothers from delivering from health facilities. Such mothers resorted to sending friends to take their babies for immunization at the health facilities as a strategy to hide from ART services and the associated stigma. In such cases, monitoring of mothers for prevention of mother to child transmission of HIV service uptake would be difficult as one of the health workers noted.

*“Our focus is mostly on babies. When positive mothers give birth out of hospital, they send friends to bring their children for immunization. Mothers remain in hiding and are not monitored for HIV. This in most cases puts babies at’ a high risk of contracting HIV from their mothers such as during breast feeding.” (Female KIs, PNC, Mubende District).*

### Too much paper work

Health workers complained of too much paper work in relation to filling registers on a daily basis. In endeavoring to provide various services by the few health workers to a multitude of mothers, some of the work would not be done. Health workers reported that due to provision of integrated services, they fill various registers including the ANC, PNC, FP, HIV, and immunization registers which is too much paper work on a daily basis. This also compromises care for the women and their children.

*“We are expected to provide various services to mothers. Each day, we try to attend to all mothers who come for ANC and by the time we close our ANC units, we are all too exhausted to fill registers. We are over whelmed … it requires a lot of time to fill different registers…. I wish the registers were also integrated…” (Female KI, ANC, Katakwi District).*

### Limited space

The limited space in health facilities was mentioned by majority of the health workers and mothers. It was noted that several additional services were introduced but the space was never adjusted which resulted into squeezing all clients in one place including those with tuberculosis.

Due to limitations in space, some of the health facilities could not provide integrated services. This was mainly attributed to few rooms. Providers reported that many mothers missed out on HCT services as a result of this limited integration. The health workers were also unable to provide various services on the same day as recommended in the integration model, and mothers were given appointments for other services on different days.

*“For us here we do not provide ANC, PNC with HIV care services on the same day. Mothers attending our health facility for ANC receive only ANC services and then they are given appointments to return to the health center for HCT and care” (Male KI, Mubende District).*

Failure to provide integrated services at certain health facilities was also confirmed by mothers during the FGDs. Mothers mentioned that HIV services were provided to mothers who requested for them and they had a separate day to visit the ART clinic. This approach caused some of the mothers to miss HIV testing due to long distances to health facilities and transport costs.

*“It becomes hard for us to return to the health facility when it is not a day for ANC. Some of us travel long distances seeking ANC services and it is hard to find transport. How can I ask my husband for transport to come to do an HIV blood test…? It is not possible…” (FGD, ANC, Mubende District).*

Limited space also led to mixing young and old mothers in ANC and PNC units making the environment unfriendly for the delivery of integrated ANC, PNC and HIV services. Services that were provided to old and young mothers in the same room and at the same time were; health education talks, ANC and PNC services. Mixing of the two groups limited the young mothers from asking questions because of fear of being teased by the older women. The challenge of mixing young and old mothers in the delivery of integrated services was also noted by mothers as illustrated in the following quotation.

*“We do not feel comfortable during health education and service provision because we are mixed with older women. This makes us fear asking questions for fear to be laughed at by the experienced women.” (FGD, ANC, Katakwi District).*

### Shortage of critical supplies

Stock out of critical supplies for integrated services was a challenge across all the health facilities and districts. Some of the supplies that were often out of stock were; HIV test kits, gloves and drugs. Limited health supplies resulted into provision of a few of the recommended services.

*“We receive 100 pairs of gloves monthly yet over 150 mothers deliver at our health facility monthly. And according to guidelines for delivering mothers, every mother requires at least 3 pairs of gloves….” (KI,Mubende District hospital).*

*…in addition, we are given few test kits compared to the high number of mothers requiring HIV testing. Sometimes mothers come and we do not have HIV testing kits so they go without being tested…..” (KI, health facility, Katakwi District).*

### Negative attitude of health workers towards additional work

Most health care providers viewed integration as additional work. Administrators at general hospitals and health center IVs reported that health workers had a negative attitude towards “added work”. District health managers and service providers noted that with introduction of the integrated services, health workers expected additional allowances over and above their monthly salaries and were hesitant to provide the integrated services as recommended in the model.

*“Health workers expected additional allowances in terms of appreciation with additional work load. This was because the few health workers available were expected to deliver a range of services to all mothers. These services were included in the work plans for the health facilities, however, health workers failed to follow the work plans, which could be attributed to poor remuneration.” (KI, Mubende District).*

### Lack of accommodation and transport for health workers

Health workers experienced challenges with transport and accommodation. Lack of accommodation within or near the health facilities resulted into additional expenditure such as high transport costs. In addition, health care providers noted that commuting from far led to late coming and delays in service provision thus negatively affecting the delivery of integrated services.

### Limited funding of some components of the services

Other challenges mentioned by health workers included limited or no funding for certain services such as the community services provided by the village health teams (VHTs). Services that were mostly provided by VHTs included home visits, identifying and referring mothers for health care services, among others. Limited funding implied limited home visitation, less or none monitoring visits that were conducted and failure to refer mothers for health care services as would be required. Limited funding also led to stock out of key supplies which hindered the delivery of integrated services.

## Discussion

This study documented the challenges faced by frontline health workers in providing integrated HIV, ANC and PNC services in two rural Ugandan Districts. The major challenges identified were few health workers and absenteeism, knowledge gaps among health workers especially with regard to provision of long-term family planning and HCT services. Other challenges included limited space for service delivery, stock out of critical supplies, high expectations of mothers and too much paperwork/many registers.

Few staff compared to the heavy work load of the health workers was a major challenge. The limited numbers of health workers led to staff burn-out, mothers delaying at health facilities and failure by health workers to provide mothers with comprehensive services. Indeed at some health facilities, vital measurements were irregular while health education talks were rushed. Our findings are consistent with those of a Kenyan study where heavy workload and less quality time with clients were key challenges to integrated HIV and SRH service provision [15]. The implication is that while service integration is critical in low income settings, matching staffing needs to the work load remains a critical gap. In Uganda, these findings are not surprising given the wide spread human resource gaps, with only 58 % of the staff positions in the public health sector filled in 2012 [16, 17]. Absenteeism of health workers and delayed salaries further exacerbated the staffing challenges.

Expectation of additional payments or incentives by health workers is a challenge that has been reported across various programs and studies that have attempted to expand or introduce new interventions to existing services [18]. These expectations are partly rooted into the challenges with human resources including limited numbers and skills, and low remuneration, among others and may require more comprehensive health systems strengthening interventions [19]. Studies on integration in other African settings have also highlighted poor remuneration of health workers as an impediment to effective integration [15, 20].

Further, some health workers lacked knowledge particularly in HIV counseling and administering long term FP methods. These findings reflect a need for refresher or targeted training to address gaps as additional services are introduced or integrated [20]. Limited skills of staff defeat the purpose of integration as the mothers continue to miss some services. For integration to take root, capacity building for service providers and managers, including coordination, leadership, and advocacy is necessary [18].

Upholding confidentiality particularly for mothers living with HIV was another critical challenge. The issue of separating HIV infected women from the HIV negative was quite prominent. Concerns about the need for confidentiality and avoidance of inadvertent disclosure of their HIV status during the provision of HIV counseling and testing services have been documented in other African settings [21, 22]. In part, failure to ensure confidentiality at some health facilities in our study was attributed to limited space for service provision [23]. Expanding space could help address this challenge to a certain extent. However, with some creativity, providers could also be able to ensure confidentiality even under difficult circumstances (limited space); for example providers have introduced approaches such as “whisper method” among others, to ensure confidentiality [24]. It is also possible to integrate the referral processes into the private consultation sessions and to avoid mention of HIV status in open and communal spaces.

Study findings revealed that health workers were faced with stock outs of critical health supplies such as drugs, mama kits and HIV test kits. Shortage of health supplies as a barrier in delivering HIV services in the antenatal clinics has been reported by other studies in Uganda and other African countries [25]. Study findings revealed that during the provision of ANC and PNC services, there was no separation of the young mothers from the old mothers. Lack of youth friendly health as a barrier for young people and mothers to access health services including maternal health has been documented elsewhere [26]. Providers and health managers should provide private space for all mothers, including the young mothers, to raise issues that they may not feel comfortable to discuss in groups.

The findings of this study should be interpreted in light of a number of limitations. The qualitative design adopted in this study makes it difficult to quantify the challenges highlighted. However, the exploratory nature of the study facilitated an in-depth understanding of the day to day challenges faced by the health workers in providing SRH and HIV services.

## Conclusion

In conclusion, the delivery of integrated SRH and HIV services in Mubende and Katakwi Districts was constrained by multiple health system challenges including few health workers, knowledge gaps, limited space in which to provide services including ensuring confidentiality, stock out of critical supplies and lack of integrated registers. These findings reflect the broader health system challenges as stumbling blocks to the delivery of quality health care in general and integration in particular. For the integration model to deliver the intended benefits, it is imperative that additional staffing at health facilities, capacity building for health workers and health managers as well as ensuring availability of sufficient supplies to health facilities are prioritized.

## Abbreviations

AIC: Aids Information Center
AIDS: Acquired Immune Deficiency Syndrome
ANC: antenatal care
ART: antiretroviral therapy
DHO: District Health Officer
FCI: Family Care International
FP: family planning
GBV: gender based violence
HCT: HIV counseling and testing
HIV: Human Immunodeficiency Virus
HW: health workers
ICPD: International Conference on Population and Development
IPPF: International Planned Parenthood Federation
MOH: Ministry of Health
OIs: opportunistic infections
PLHIV: persons living with HIV
PMTCT: prevention of mother to child transmission
SRH: sexual and reproductive health
SRHS: Sexual and Reproductive Health Services
STI: sexually transmitted infections
TB: tuberculosis
UNAIDS: United Nations Program on HIV/AIDS
UNFPA: United Nations Population Fund
VCT: Voluntary Counseling and Testing
WHO: World Health Organization
